# Preterm Deliveries in Women with Uterine Myomas: The Japan Environment and Children’s Study

**DOI:** 10.3390/ijerph18052246

**Published:** 2021-02-24

**Authors:** Tsuyoshi Murata, Hyo Kyozuka, Yuta Endo, Toma Fukuda, Shun Yasuda, Akiko Yamaguchi, Akiko Sato, Yuka Ogata, Kosei Shinoki, Mitsuaki Hosoya, Seiji Yasumura, Koichi Hashimoto, Hidekazu Nishigori, Keiya Fujimori, The Japan Environment and Children’s Study (JECS) Group

**Affiliations:** 1 Fukushima Regional Center for the Japan Environment and Children’s Study, 1 Hikarigaoka, Fukushima 960-1295, Japan; kyozuka@fmu.ac.jp (H.K.); yenyen@fmu.ac.jp (Y.E.); t323@fmu.ac.jp (T.F.); room335@fmu.ac.jp (S.Y.); akiko-y@fmu.ac.jp (A.Y.); asato@fmu.ac.jp (A.S.); yuka-o@fmu.ac.jp (Y.O.); shinok46@fmu.ac.jp (K.S.); mhosoya@fmu.ac.jp (M.H.); yasumura@fmu.ac.jp (S.Y.); don@fmu.ac.jp (K.H.); nishigo@fmu.ac.jp (H.N.); fujimori@fmu.ac.jp (K.F.); 2 Department of Obstetrics and Gynecology, Fukushima Medical University School of Medicine, 1 Hikarigaoka, Fukushima 960-1295, Japan; 3 Department of Pediatrics, Fukushima Medical University School of Medicine, 1 Hikarigaoka, Fukushima 960-1295, Japan; 4 Department of Public Health, Fukushima Medical University School of Medicine, 1 Hikarigaoka, Fukushima 960-1295, Japan; 5 Fukushima Medical Center for Children and Women, Fukushima Medical University, 1 Hikarigaoka, Fukushima 960-1295, Japan

**Keywords:** birth-cohort study, uterine myoma, preterm birth, preterm premature rupture of membranes, intrauterine infection

## Abstract

This study aimed to clarify the association between uterine myomas and preterm birth (PTB), preterm premature rupture of membranes (pPROM), and intrauterine infection (II). The study was based on data from the Japan Environment and Children’s Study, a nationwide birth-cohort study. Data of 86,370 women with singleton births after 22 weeks of gestation (with uterine myomas, *n* = 5354) were retrospectively analyzed. Using logistic regression, adjusted odds ratios (aORs) for PTB, pPROM, and II were calculated considering women without uterine myomas as the reference. Additionally, the effects of II on the incidence of PTB and pPROM were evaluated. In women with uterine myomas, the aORs for PTB before 37 and 34 weeks, pPROM, and II were 1.37 (95% confidence interval, 1.22–1.54), 1.61 (1.27–2.05), 1.65 (1.33–2.04), and 1.05 (0.75–1.46), respectively. The aORs for PTB and pPROM in women with II and uterine myomas were not significantly increased. Uterine myomas during pregnancy were associated with an increased incidence of PTB and pPROM. However, II in women with uterine myomas was not associated with an increased incidence of PTB or pPROM. These findings suggest a potential risk of occult PTB in pregnant women with uterine myomas.

## 1. Introduction

Uterine myomas (also known as leiomyomata, fibroids, fibromyomas, leiomyofibromas, and fibroleiomyomas) are benign tumors that originate from clonal proliferation of smooth muscle cells of the uterus; they are common among women of reproductive age, with a prevalence of 20–60% [1,2]. The prevalence of uterine myomas in pregnant women has been reported as 0.4–10.7%; however, there is conflicting evidence regarding the obstetric outcomes in this group [1,2]. Although some studies have reported no increased risk of adverse pregnancy outcomes [3,4,5], other studies have found that uterine myomas during pregnancy increase the risk of preterm birth (PTB) [1,2,6,7].

In children, PTB accounts for more than 70% of perinatal mortalities and more than half of long-term morbidities [8,9,10]. Thus, PTB prevention with regard to particular causal factors is paramount for improving neonatal outcomes; moreover, detailed evaluation of women at risk for PTB is required for tailored treatment [10]. Although a major etiology of spontaneous PTB is premature labor triggered by an unknown cause [8], preterm premature rupture of membranes (pPROM), which is often followed by intrauterine infection (II), is a risk factor associated with PTB [11]. Therefore, accurate assessment of risk factors for PTB and its causal factors in women with uterine myomas is important for patients and physicians because of the frequency of uterine myomas in pregnant women.

However, the biochemical mechanisms underlying PTB among women with uterine myomas, as well as the effects of uterine myomas on the course of pregnancy, remain unclear [1,2,6]. This is partially because the majority of previous studies on the risks of PTB in women with uterine myomas were retrospective and involved small samples [5,12], yielding conflicting results on the association between uterine myomas and PTB, pPROM, and II [1,2,3,4,5,6,13]. Therefore, in the present study, we aimed to clarify the association of uterine myomas during pregnancy with PTB, pPROM, and II relative to the general population, and to evaluate the effects of II on the incidence of PTB and pPROM in women with uterine myomas, using data from a nationwide Japanese birth-cohort study.

## 2. Materials and Methods

### 2.1. Study Design

This was a retrospective analysis based on data from the Japan Environment and Children’s Study (JECS), which is a government-funded prospective birth-cohort study launched in January 2011 to investigate the effects of environmental factors on children’s health [14,15]. Briefly, the JECS is funded by the Ministry of the Environment, Japan and involves the collaboration between the Programme Office (National Institute for Environmental Studies), Medical Support Center (National Center for Child Health and Development), and 15 Regional Centers (Hokkaido, Miyagi, Fukushima, Chiba, Kanagawa, Koshin, Toyama, Aichi, Kyoto, Osaka, Hyogo, Tottori, Kochi, Fukuoka, and South Kyushu/Okinawa) [15]. For inclusion as the JECS participants, expectant mothers had to meet the following criteria: (1) residence within the study area at the time of recruitment and expectation to continue residing in Japan for the foreseeable future; (2) expected due date between 1 August 2011 and mid-2014; and (3) capacity to participate in the study without difficulty (i.e., ability to comprehend the Japanese language and complete a self-administered questionnaire).

There were two modes of recruitment: (1) at the time of the first prenatal examination at participating obstetric facilities; and (2) at local government offices issuing a pregnancy journal, called the Maternal and Child Health Handbook, given to all expecting mothers before they receive municipal services for pregnancy, delivery, and childcare in Japan. We contacted pregnant women through cooperating health care providers and/or local government offices issuing Maternal and Child Health Handbooks and registered those willing to participate. Self-administered questionnaires, which were completed by the women during the first and the second/third trimester, were used to collect information on demographic factors, medical history, physical and mental health, lifestyle, occupation, environmental exposure at home and in the workplace, housing conditions, and socioeconomic status [15].

### 2.2. Data Collection

The present analysis used data released in June 2016 (data set: JECS-ag-20160424). Specifically, we used three types of data: (1) M-T1, which are data on maternal medical background, obtained using a self-reported questionnaire during the first trimester (the first questionnaire); (2) M-T2, which are data on socioeconomic status, obtained with a self-reported questionnaire during the second or third trimester (second questionnaire); and (3) Dr-0m, which are data on obstetric outcomes, such as gestational age and birth weight, collected throughout pregnancy and extracted from participants’ medical record transcripts (provided by relevant institutions).

Participants with singleton pregnancies after 22 weeks of gestation were included in the present analysis. Women who underwent an abortion, experienced a stillbirth, and those with missing information were excluded from the analysis. There were no statistically significant differences in characteristics between those included and those excluded from the analysis (data not shown).

### 2.3. Exposure Variables

Women with uterine myomas were identified based on a self-reported questionnaire. Uterine myomas were diagnosed before pregnancy or at the first trimester through ultrasonography. The JECS only included information regarding the presence or absence of uterine myomas, and therefore, no information about number, size, and localization of uterine myomas was available. Participants with uterine myomas were categorized into the myoma group and those without uterine myomas were categorized into the reference group.

### 2.4. Obstetric Outcomes and Confounding Factors

PTBs were categorized into two groups, before 37 and before 34 weeks, because infants born before 34 weeks require antenatal corticosteroid therapy for fetal maturation. pPROM was defined as spontaneous rupture of membranes before 37 weeks. II information was derived from medical record transcripts. II was clinically diagnosed by physicians at each institution. There were no unified criteria for II in the JECS; however, most Japanese obstetricians refer to the criteria recommended in the guidelines for obstetrical practice in Japan, i.e., maternal fever and one of the following: maternal tachycardia beyond 100 beats/min, uterine tenderness, abnormal discharge, or maternal white blood cell counts beyond 15,000/µL [16]. Histological findings for chorioamnionitis were not required for the diagnosis of II in the JECS.

The following items were considered potential confounding factors: maternal age, maternal body mass index (BMI) before pregnancy, parity, maternal smoking status, maternal educational status, and annual household income. Participants were classified into three groups based on maternal age: <20, 20–34, and ≥35 years [17]. Participants were categorized into three groups based on pre-pregnancy BMI: <18.5, 18.5–25.0, and ≥25.0 kg/m^2^. Participants were categorized into two groups based on parity: nulliparous and multiparous. Participants were requested to provide information regarding their smoking status by choosing one of the following: “Currently smoking,” “Never,” “Previously did, but quit before realizing current pregnancy,” and “Previously did, but quit after realizing current pregnancy.” Participants who chose “Currently smoking” and those who did not were included in the “smoking” and “non-smoking” categories, respectively. Participants were categorized into four groups based on their completed number of years of education (junior high school, <10 years; high school, 10–12 years; technical/vocational school or university, 13–16 years; and graduate school, ≥17 years). Participants were categorized into four groups based on their annual household income (<2,000,000, 2,000,000–5,999,999, 6,000,000–9,999,999, and ≥10,000,000 JPY). These confounding factors were chosen based on clinical importance [18,19].

### 2.5. Statistical Analysis

Participants were stratified based on the presence of uterine myomas; clinical and demographic sample characteristics were reported accordingly. Student’s *t*-test and Mann-Whitney U test were used to compare continuous variables between the groups, according to the difference of the distribution of data. The chi-square test was used to compare categorical variables between the groups. First, crude odds ratios (cORs), adjusted odds ratios (aORs), and 95% confidence intervals (CIs) for PTB, pPROM, and II were calculated using a multiple logistic regression model with the reference group as the reference. Second, the association between II and the incidence of PTB and pPROM in women with uterine myomas was evaluated using a multiple logistic regression model with women with uterine myomas without II used as the reference. Additionally, the association between II and the incidence of PTB and pPROM in women without uterine myomas was evaluated using a multiple logistic regression model with women without either uterine myomas or II used as the reference. All odds ratios (ORs) were adjusted for maternal age, maternal BMI before pregnancy, parity, maternal smoking status, maternal educational status, and annual household income.

SPSS version 26 (IBM Corp., Armonk, NY, USA) was used for statistical analysis. A *p*-value < 0.05 indicated statistical significance.

### 2.6. Ethical Approval

The JECS protocol was reviewed and approved by the Ministry of the Environment Institutional Review Board on Epidemiological Studies (No.100910001) and the ethics committees of all participating institutions. The JECS was conducted in accordance with the Helsinki Declaration and other national regulations and guidelines. Written informed consent was obtained from all participants.

## 3. Results

**Figure 1:**
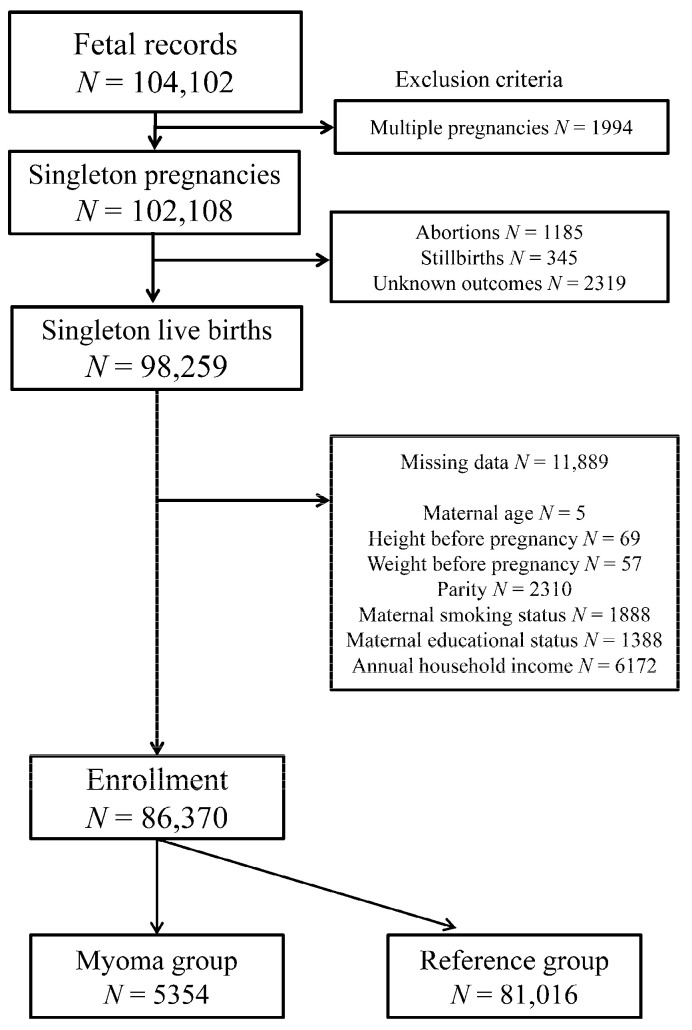
Flow chart depicting participant eligibility for study enrollment.

The total number of fetal records during 2011–2014 in the JECS was 104,102. A total of 86,370 participants met the inclusion criteria (Figure 1). Among them, 5354 participants were enrolled into the myoma group, while the remaining 81,016 were enrolled into the reference group.

Table 1 summarizes the clinical and demographic characteristics and obstetric outcomes. The incidence of PTB and pPROM was higher in the myoma group than in the reference group. 

The cORs and aORs are summarized in Table 2. The aORs for PTB before 37 and 34 weeks, pPROM, and II in the myoma group were 1.37 (95% CI, 1.22–1.54), 1.61 (95% CI, 1.27–2.05), 1.65 (95% CI, 1.33–2.04), and 1.05 (95% CI, 0.75–1.46), respectively. The aORs for PTB and pPROM were significantly increased in the myoma group.

The association between II and the incidence of PTB and pPROM in women with and without uterine myomas is summarized in Table 3. The aORs for PTB and pPROM in women with both II and uterine myomas were 1.81 (95% CI, 0.64–5.15) and 1.29 (95% CI, 0.17–9.54), respectively, while the aORs for PTB and pPROM in women with II without uterine myomas were 3.16 (95% CI, 2.39–4.19) and 5.54 (95% CI, 3.72–8.25), respectively. Although the aORs for PTB and pPROM were significantly increased in women with II without uterine myomas, the aORs were not significantly increased in women with both II and uterine myomas.

## 4. Discussion

### 4.1. Main Findings

In the present study, there was a higher incidence of PTB and pPROM among pregnant women with uterine myomas than among those without uterine myomas. However, no statistically significant association between uterine myomas during pregnancy and II was observed. In addition, II was not associated with an increased incidence of PTB and pPROM in women with uterine myomas, contrary to the results in the general population.

### 4.2. Interpretation

Uterine myomas during pregnancy were associated with an increased incidence of PTB, which is consistent with findings of recent studies [1,2,6,7]. A previous retrospective cohort study, in which the prevalence of uterine myomas was approximately 3%, found that uterine myomas during pregnancy were significantly associated with an increased incidence of PTB before 37 weeks (aOR, 1.5; 95% CI, 1.3–1.8) and 34 weeks (aOR, 1.4; 95% CI, 1.0–1.8) [1]. However, another retrospective cohort study, in which the prevalence of uterine myomas was approximately 1.5% and the analyses were based on only 183 women with uterine myomas, showed no such association [13]. This study was based on a sample of 5354 women with uterine myomas. The prevalence of uterine myomas was 6%, which was higher than that in the two abovementioned reports, but within the range of 0.4–10.7% reported in recent studies [1,2]. Overall, these findings support the hypothesis that uterine myomas during pregnancy are associated with an increased incidence of PTB.

There was an association between uterine myomas during pregnancy and an increased incidence of pPROM. Previous studies on this association showed conflicting results [2,3,4,5,6,13]. One retrospective cohort study reported a higher incidence of pPROM (aOR, 1.3; 95% CI, 1.0–1.7) [1]; our findings were consistent with those of that study. As pPROM precedes 40–50% of PTB cases [20,21], it is also likely to induce PTB in women with uterine myomas. As the rate of neonatal mortality and morbidity associated with PTB is higher in groups affected by pPROM than in any other subgroup of PTB [21], pPROM is likely to increase the potential risk of adverse neonatal outcomes in women with uterine myomas. These findings suggested that obstetricians should communicate the fact that uterine myomas during pregnancy may be associated with an increased incidence of pPROM and PTB to pregnant women.

Compared with the reference group, we found no increased incidence of II in the myoma group; additionally, II was not associated with an increased incidence of PTB or pPROM in the myoma group. To the best of our knowledge, no study to date has shown any association between uterine myomas and an increased incidence of II in pregnancy. Previous studies have shown II to be a major risk factor for PTB [8,11] and pPROM prior to labor [20]. Moreover, microbiological studies have suggested that II might account for 25–40% of PTB cases, while approximately 70% of pPROM cases were associated with II [8,11]. This study showed that II might have been a risk factor for PTB and pPROM in the reference group, but not in the myoma group. Although the sample size of participants with II was small, and although there were ambiguities regarding the classification and diagnosis of II owing to the confusion regarding its clinical characteristics, biomarkers, and pathological findings [22], the present study is the first large-cohort analysis to evaluate the association between uterine myomas during pregnancy and II diagnosed clinically.

The present findings suggest a characteristic etiology of PTB and pPROM in women with uterine myomas. Although the biological mechanism of PTB in pregnant women with uterine myomas remains unclear [1,6], studies have shown that spontaneous PTB, including pPROM in cases with uterine myomas, was associated with distortion of the uterine cavity and loss of uterine distensibility [23,24]. In addition, hormonal changes have been implicated in spontaneous PTB [2]. Uterine myomas may compromise the myometrium and cause decidualization of endometrial stromal fibroblasts, inducing spontaneous PTB, as is the case in endometriosis [2,25,26]. Therefore, the present findings may support the notion of a mechanical effect of uterine myomas rather than one depending on inflammatory factors in PTB and pPROM. PTB and pPROM without II may lack clinical features and may lead to incidences of occult PTB and pPROM in pregnant women with uterine myomas.

### 4.3. Strengths and Limitations

A major strength of the present study is the inclusion of a large sample of a nationwide cohort study that yielded robust findings regarding the association between uterine myomas and obstetric outcomes. Specifically, the aOR for PTB before 34 weeks in women with uterine myomas was nearly twice that in the reference group, suggesting that uterine myomas during pregnancy may be directly linked to neonatal outcomes. The present findings suggested the need to appropriately evaluate the PTB risk and manage pregnancies in women with uterine myomas, e.g., by administration of vaginal progesterone, antenatal corticosteroids, antibiotics, and magnesium sulfate [27,28,29]. This may reduce PTBs and improve neonatal outcomes.

This study had several limitations. First, information regarding uterine myomas was based on a self-reported questionnaire. Additionally, the protocol for diagnosing uterine myomas was not unified, and variations in their number, size, and site, as well as the treatment history for uterine myomas before and during pregnancy (including hormonal treatments and myomectomy) were not considered. As these factors may affect obstetric outcomes [2,18,19], future studies should include evaluations of these factors. Second, several maternal characteristics previously associated with PTB were not considered (i.e., certain demographic and psychological characteristics, detailed history of PTB, adverse behaviors, drug abuse, uterine contractions, cervical length, and biological and genetic markers). These implicated factors for PTB [8] should be evaluated in future studies. Nevertheless, we included a large sample and accounted for factors such as maternal smoking status, maternal educational status, and annual household income, all of which contribute to the robustness of the present findings. Third, II was diagnosed based on clinical features, rather than histological findings. Histological analysis of II may strengthen the results of the present analysis. Further studies including histological evaluation for II are required to clarify the association between uterine myomas during pregnancies and II. Lastly, evaluation of uterine myomas during pregnancy might contribute to potential bias. Therefore, the findings of our study should be carefully interpreted, and further studies that use strategies such as matching or weighting to eliminate potential bias should be conducted.

## 5. Conclusions

Uterine myomas during pregnancy were associated with an increased incidence of PTB and pPROM. Moreover, PTB and pPROM were not associated with II in women with uterine myomas. These findings suggest a potential risk of occult PTB and pPROM in pregnant women with uterine myomas. Obstetricians should counsel their patients regarding the potential risks associated with uterine myomas in pregnancy and offer suitable interventions to prevent and manage PTB and pPROM. 

## Figures and Tables

**Table 1:** Demographic and clinical characteristics and obstetric outcomes of participants stratified according to the uterine myoma status.

Variable	Total Participants (*n* = 86,370)	Myoma Group (*n* = 5354)	Reference Group (*n* = 81,016)	*p*-Value
Maternal age, years mean (*SD*)	31.3 (4.9)	35.1 (4.1)	31.1 (4.9)	<0.001
Maternal BMI before pregnancy, kg/m^2^ mean (*SD*)	21.2 (3.3)	21.9 (3.5)	21.2 (3.3)	<0.001
Nulliparous, % (*n*)	39.9 (34,478)	47.9 (2563)	39.4 (31,915)	<0.001
Maternal smoking during pregnancy, % (*n*)	4.7 (4036)	3.0 (162)	4.8 (3874)	<0.001
Maternal educational status, % (*n*)				
<10 years	4.5 (3909)	2.1 (112)	4.7 (3797)	<0.001
10–12 years	30.8 (26,573)	24.8 (1328)	31.2 (25,245)	<0.001
13–16 years	63.2 (54,586)	70.7 (3783)	62.7 (50,803)	<0.001
≥17 years	1.5 (1302)	2.4 (131)	1.4 (1171)	<0.001
Annual household income, % (*n*)				
<2,000,000 JPY	5.6 (4878)	2.8 (151)	5.8 (4727)	<0.001
2,000,000–5,999,999 JPY	67.6 (58,392)	62.3 (3336)	68.0 (55,056)	<0.001
6,000,000–9,999,999 JPY	22.5 (19,404)	28.6 (1530)	22.1 (17,874)	<0.001
≥10,000,000 JPY	4.3 (3696)	6.3 (337)	4.1 (3359)	<0.001
PTB <37 weeks, % (*n*)	4.5 (3901)	6.2 (333)	4.4 (3568)	<0.001
PTB <34 weeks, % (*n*)	0.9 (752)	1.5 (79)	0.8 (673)	<0.001
pPROM, % (*n*)	1.1 (959)	1.8 (98)	1.1 (861)	<0.001
II, % (*n*)	0.6 (493)	0.7 (39)	0.6 (454)	0.114

SD, standard deviation; BMI, body mass index; PTB, preterm birth; pPROM, preterm premature rupture of membranes; II, intrauterine infection.

**Table 2:** Odds ratios for obstetric complications in the myoma group.

Obstetric Outcomes	PTB	PTB	pPROM	II
	<37 Weeks	<34 Weeks		
Myoma Group	Odds Ratios (95% CI)			
cORs	1.44 (1.28–1.62)	1.79 (1.41–2.26)	1.74 (1.41–2.14)	1.30 (0.94–1.81)
aORs	1.37 (1.22–1.54)	1.61 (1.27–2.05)	1.65 (1.33–2.04)	1.05 (0.75–1.46)

cOR, crude odds ratio; aOR, adjusted odds ratio; CI, confidence interval; PTB, preterm birth; pPROM, preterm premature rupture of membranes; II, intrauterine infection. The multivariate logistic regression analyses were adjusted for maternal age, maternal body mass index before pregnancy, parity, maternal smoking status, maternal educational status, and annual household income.

**Table 3:** Adjusted odds ratios for PTB and pPROM in women with II.

Obstetric Outcomes	Myoma Group		Reference Group	
	PTB	pPROM	PTB	pPROM
aORs				
II (–)	Ref	Ref	Ref	Ref
II (+)	1.81 (0.64–5.15)	1.29 (0.17–9.54)	3.16 (2.39–4.19)	5.54 (3.72–8.25)

aOR, adjusted odds ratio; PTB, preterm birth; pPROM, preterm premature rupture of membrane; II, intrauterine infection; Ref, reference. The multivariate logistic regression analyses were adjusted for maternal age, maternal body mass index before pregnancy, parity, maternal smoking status, maternal educational status, and annual household income.

## Data Availability

Data generated in this study are unsuitable for public deposition due to ethical restrictions and the legal framework of Japan. It is prohibited by the Act on the Protection of Personal Information (Act No. 57 of 30 May 2003, amendment on 9 September 2015) to publicly deposit data containing personal information. Ethical Guidelines for Epidemiological Research enforced by the Japan Ministry of Education, Culture, Sports, Science and Technology and the Ministry of Health, Labour and Welfare also restricts the open sharing of the epidemiologic data. All inquiries about access to data should be sent to: jecs-en@nies.go.jp. The person responsible for handling enquiries sent to this e-mail address is Dr Shoji F. Nakayama, JECS Programme Office, National Institute for Environmental Studies.

## References

[1] Stout M.J., Odibo A.O., Graseck A.S., Macones G.A., Crane J.P., Cahill A.G. (2010). Leiomyomas at routine second-trimester ultrasound examination and adverse obstetric outcomes. Obstet. Gynecol..

[2] Girault A., Le Ray C., Chapron C., Goffinet F., Marcellin L. (2018). Leiomyomatous uterus and preterm birth: An exposed/unexposed monocentric cohort study. Am. J. Obstet. Gynecol..

[3] Coronado G.D., Marshall L.M., Schwartz S.M. (2000). Complications in pregnancy, labor, and delivery with uterine leiomyomas: A population-based study. Obstet. Gynecol..

[4] Exacoustòs C., Rosati P. (1993). Ultrasound diagnosis of uterine myomas and complications in pregnancy. Obstet. Gynecol..

[5] Davis J.L., Ray-Mazumder S., Hobel C.J., Baley K., Sassoon D. (1990). Uterine leiomyomas in pregnancy: A prospective study. Obstet. Gynecol..

[6] Qidwai G.I., Caughey A.B., Jacoby A.F. (2006). Obstetric outcomes in women with sonographically identified uterine leiomyomata. Obstet. Gynecol..

[7] Chen Y.H., Lin H.C., Chen S.F., Lin H.C. (2009). Increased risk of preterm births among women with uterine leiomyoma: A nationwide population-based study. Hum. Reprod..

[8] Goldenberg R.L., Culhane J.F., Iams J.D., Romero R. (2008). Epidemiology and causes of preterm birth. Lancet.

[9] Wen S.W., Smith G., Yang Q., Walker M. (2004). Epidemiology of preterm birth and neonatal outcome. Semin. Fetal Neonatal Med..

[10] López Bernal A. (2007). Overview. Preterm labour: Mechanisms and management. BMC Pregnancy Childbirth.

[11] Ananth C.V., Vintzileos A.M. (2006). Epidemiology of preterm birth and its clinical subtypes. J. Matern. Fetal Neonatal Med..

[12] Rice J.P., Kay H.H., Mahony B.S. (1989). The clinical significance of uterine leiomyomas in pregnancy. Am. J. Obstet. Gynecol..

[13] Vergani P., Ghidini A., Strobelt N., Roncaglia N., Locatelli A., Lapinski R.H., Mangioni C. (1994). Do uterine leiomyomas influence pregnancy outcome?. Am. J. Perinatol..

[14] Kawamoto T., Nitta H., Murata K., Toda E., Tsukamoto N., Hasegawa M., Yamagata Z., Kayama F., Kishi R., Ohya Y. (2014). Rationale and study design of the Japan Environment and Children’s Study (JECS). BMC Public Health.

[15] Michikawa T., Nitta H., Nakayama S.F., Yamazaki S., Isobe T., Tamura K., Suda E., Ono M., Yonemoto J., Iwai-Shimada M. (2018). Baseline profile of participants in the Japan Environment and Children’s Study (JECS). J. Epidemiol..

[16] Lencki S.G., Maciulla M.B., Eglinton G.S. (1994). Maternal and umbilical cord serum interleukin levels in preterm labor with clinical chorioamnionitis. Am. J. Obstet. Gynecol..

[17] Kyozuka H., Fujimori K., Hosoya M., Yasumura S., Yokoyama T., Sato A., Hashimoto K., Japan Environment and Children’s Study (JECS) Group (2019). The effect of maternal age at the first childbirth on gestational age and birth weight: The Japan Environment and Children’s Study (JECS). J. Epidemiol..

[18] Ancel P.Y. (2002). Preterm labor: Pathophysiology, risk factors and outcomes. J. Gynecol. Obstet. Biol. Reprod..

[19] Goldenberg R.L., Goepfert A.R., Ramsey P.S. (2005). Biochemical markers for the prediction of preterm birth. Am. J. Obstet. Gynecol..

[20] Naeye R.L., Peters E.C. (1980). Causes and consequences of premature rupture of fetal membranes. Lancet.

[21] Menon R., Richardson L.S. (2017). Preterm prelabor rupture of the membranes: A disease of the fetal membranes. Semin. Perinatol..

[22] Higgins R.D., Saade G., Polin R.A., Grobman W.A., Buhimschi I.A., Watterberg K., Silver R.M., Raju T.N.K., Chorioamnionitis Workshop Participants (2016). Evaluation and management of women and newborns with a maternal diagnosis of chorioamnionitis: Summary of a workshop. Obstet. Gynecol..

[23] Roberts D., Brown J., Medley N., Dalziel S.R. (2017). Antenatal corticosteroids for accelerating fetal lung maturation for women at risk of preterm birth. Cochrane Database Syst. Rev..

[24] Jarde A., Lutsiv O., Beyene J., McDonald S.D. (2019). Vaginal progesterone, oral progesterone, 17-OHPC, cerclage, and pessary for preventing preterm birth in at-risk singleton pregnancies: An updated systematic review and network meta-analysis. BJOG.

[25] Duley L., Gülmezoglu A.M., Henderson-Smart D.J., Chou D. (2010). Magnesium sulphate and other anticonvulsants for women with pre-eclampsia. Cochrane Database Syst. Rev..

[26] Ciavattini A., Clemente N., Delli Carpini G., Di Giuseppe J., Giannubilo S.R., Tranquilli A.L. (2015). Number and size of uterine fibroids and obstetric outcomes. J. Matern. Fetal Neonatal Med..

[27] Marzano S., Padula F., Meloni P., Anceschi M.M. (2008). Preterm delivery at low gestational age: Risk factors for short latency. A multivariated analysis. J. Prenat. Med..

[28] Zullo F., Spagnolo E., Saccone G., Acunzo M., Xodo S., Ceccaroni M., Berghella V. (2017). Endometriosis and obstetrics complications: A systematic review and meta-analysis. Fertil. Steril..

[29] Petraglia F., Arcuri F., de Ziegler D., Chapron C. (2012). Inflammation: A link between endometriosis and preterm birth. Fertil. Steril..

